# Potential Clinical Usefulness of Post-Valvular Contrast Densities to Determine the Severity of Aortic Valve Stenosis Using Computed Tomography

**DOI:** 10.3390/jcdd10100412

**Published:** 2023-09-30

**Authors:** Agnes Orsolya Racz, Gabor Tamas Szabo, Tamas Papp, Benjamin Csippa, Daniel Gyurki, Bertalan Kracsko, Zsolt Koszegi, Rudolf Kolozsvari

**Affiliations:** 1 Department of Cardiology and Heart Surgery, University of Debrecen, 4032 Debrecen, Hungary; racz.agnes@med.unideb.hu (A.O.R.); nszgt@med.unideb.hu (G.T.S.); kracsko.bertalan@med.unideb.hu (B.K.); koszegi@med.unideb.hu (Z.K.); 2 Department of Radiology, University of Debrecen, 4032 Debrecen, Hungary; papp.tamas@med.unideb.hu; 3 Department of Hydrodynamic Systems, University of Technology and Economics, 1111 Budapest, Hungary; bcsippa@hds.bme.hu (B.C.); dgyurki@hds.bme.hu (D.G.); 4 3rd Department of Internal Medicine, Szabolcs-Szatmar-Bereg County Hospital, 4400 Nyíregyháza, Hungary

**Keywords:** aortic valve stenosis, computed tomography, TAVR, echocardiography, contrast density

## Abstract

Background: Different methods are established for the changes in aortic valve stenosis with cardiac computed tomography angiography (CCTA), but the effect of the grade of stenosis on contrast densities around the valve has not been investigated. Aims/methods: Using the information from flow dynamics in cases of increased velocity through narrowed lumen, the hypothesis was formed that flow changes can alter the contrast densities in stenotic post-valvular regions, and the density changes might correlate with the grade of stenosis. Forty patients with severe aortic stenosis and fifteen with a normal aortic valve were enrolled. With echocardiography, the peak/mean transvalvular gradients, peak transvalvular velocity, and aortic valve opening area were obtained. With CCTA, densities 4–5 mm above the aortic valve; at the junction of the left, right, and noncoronary cusp to the annulus; at the middle level of the left, right, and noncoronary sinuses of Valsalva in the center and the lateral points; at the sinotubular junction; and 4 cm from the sinotubular junction at the midline were measured. First, a comparison of the densities between the normal and stenotic valve was performed, and then possible correlations between echocardiography and CCTA values were investigated in the stenotic group. Results: In all CCTA regions, significantly lower-density values were detected among stenotic valve patients compared to the normal aortic valve population. Additionally, in both groups, higher densities were measured in the peri-jet regions than in the lateral ones. Furthermore, a good correlation was found between the aortic valve opening area and the densities in almost all perivalvular areas. With regard to the densities at the junction of the non-coronary leaflet to the fibrotic annulus and at the most lateral point of the right sinus of Valsalva, a high level of correlation was found between all echocardiography and CCTA parameters. Lastly, with receiver operating characteristic curve measurements, area under the curve values were between 0.857 and 0.930. Conclusion: Certain CCTA density values, especially 4–5mm above the valve opening, can serve as auxiliary information to echocardiography when the severity of aortic valve stenosis is unclear.

## 1. Background

**Figure 1:**
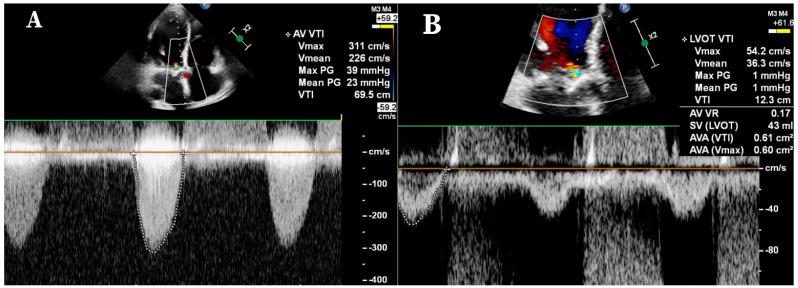
Low-flow, low-gradient aortic stenosis detected by transthoracic echocardiography. Continuous wave Doppler imaging shows low gradients and flow velocity across the calcified aortic valve. Panel (**A**): mean and peak gradient at 23 mmHg and 39 mmHg, respectively; maximum velocity at 3.11 m/s—non-significant values presented. Panel (**B**): left ventricular outflow tract velocity time integral is 12.3 cm; calculated aortic valve area is 0.61 cm^2^—significant and critical valve disease observed. AV: aortic valve; Vmax: maximum velocity; Vmean: mean velocity; VTI: velocity time integral; PG: pressure gradient; LVOT: left ventricular outflow gradient; VR: velocity ratio; SV: stroke volume; AVA: aortic valve area.

Aortic valve stenosis is a progressive disease, with senile calcification as the most common etiology [1]. The gold standard examination for defining the grade remains echocardiography (ECHO) [2]. In almost one-third of patients with an aortic valve area (AVA) ≤1 cm^2^, the maximum velocity above the opening of the aortic valve (V_max_) and the gradients do not necessarily correlate with the AVA due to the character of the V_max_ and the pressure gradients being highly flow-dependent. Figure 1 presents a low-flow, low-gradient aortic stenosis situation detected by transthoracic echocardiography, showing low gradients and flow velocity across the calcified aortic valve presuming possibly non-significant aortic stenosis, whereas the aortic valve area is significantly decreased, assuming severe aortic stenosis (SAS). The above phenomenon often causes difficulties in defining the proper time of the valve replacement, raising the need for another imaging method to properly quantify the grade of severity [3]. Recent cardiac computed tomography angiography (CCTA) examinations focusing on the stenotic/calcified aortic valve can serve as a “decision maker” when ECHO results are discordant [4,5,6].

With regard to hemodynamics, in the case of a patent aortic valve, the aortic flow is helical with end-systolic retrograde flow in areas where the kinetic energy of blood is high, usually at the greater curve of the ascending aorta, shown by both in vitro and in vivo studies [7,8]. The importance of the helical character is to optimize the flow on the aorta avoiding pathological energy disintegration and flow instabilities [9]. In the case of SAS, the increased speed of the blood above the aortic valve results in an eccentric flow, consequential wall shear stress changes, and an unphysiological helical flow pattern [10]. As a result of these hemodynamic changes, on a morphological level, ascending aorta dilation and dissection occur, whereas on a cellular level, platelet activation and valvular thrombosis can evolve [11,12].

Recently, transcatheter aortic valve replacement (TAVR) was introduced to the field as an alternative method of severe aortic valve stenosis management [13]. Prior to TAVR, it is mandatory to perform CCTA of the aortic root. Numerous perivalvular dimensions of the aortic annulus, sinus of Valsalva, and sinotubular junction, along with angiography of the entire length of the aorta and the femoral arteries, are measured to rule out any access route alterations, along with providing other evaluation/diagnostic possibilities in the perivalvular regions [14].

Our hypothesis was that the above-mentioned hemodynamic changes in SAS should influence the CCTA contrast densities in the perivalvular regions, which has never been investigated before. With the results in hand, it might be possible to offer the guideline-recommended gold standard ECHO, an auxiliary diagnostic method to evaluate the severity of aortic stenosis in cases in which its significance cannot be defined exactly.

## 2. Methods

All patients were first seen in the Outpatient Clinic of the Department of Cardiology and Heart Surgery, University of Debrecen, Hungary, where demographic data collection and physical examination along with ECHO were performed. In the case of SAS as defined below, CCTA was performed, and these patients created group 1. Patients with non-SAS were selected as not having the criteria for SAS but had previously undergone CCTA for coronary evaluation based on the indication of chronic coronary disease, belonging to group 2. Both ECHO and CCTA had to have been performed within six months.

Patients with severe aortic stenosis AVA ≤ 1 cm^2^ by ECHO, aged > 18, and with pre-TAVR CCTA performed met the inclusion criteria for group 1. Exclusion criteria were defined as not consenting for CCTA and having a bicuspid aortic valve defined by ECHO, as well as aged < 18. Low-flow, low-gradient cases were excluded from the study. For group 2, inclusion and exclusion criteria were the same as for group 1, except for AVA being >1 cm^2^ and peak transvalvular velocity ≤ 2.5 m/s. Based on the above, 40 and 15 patients were enrolled in group 1 and 2, respectively.

The severity of aortic stenosis was evaluated by the standard ECHO measurements [15]. The continuity equation was used to define AVA as follows:

$$AVA=\frac{LVOT\;VTIx\pi x{\left(LVOTd/2\right)}^{2}}{AoVVTI}$$

where LVOT VTI represents the left ventricular outflow tract velocity time integral; LVOTd is the left ventricular outflow tract diameter; and AoVVTI is the aortic valve velocity time integral. The peak and mean gradients (PG/MG) were obtained by continuous wave Doppler. The grade of aortic regurgitation was defined by visual evaluation (0–4), whereas the left ventricular ejection fraction (LVEF) was defined by the Simpson biplane method.

Every CCTA was performed with a GE Lightspeed 64-detector VCT (GE Healthcare, Boston, MA, USA) with the helical mode in the retrospective ECG-triggered mode, a tube voltage of 100 kV, with the current adjusted automatically, and a slice thickness of 0.625 mm. Omnipaque 350 mg/mL (GE Healthcare, Boston, MA, USA) contrast media (40 mL with 2.5 mL/s) and normal saline (50 mL with 5 mL/s) via an 18G line placed in the right middle antecubital vena were administered for groups 1 and 2, respectively. Image reconstruction was conducted at a 20% RR interval as the highest flow through the most widely opened valve is approximately 200 msec after the start of the QRS.

**Figure 2:**
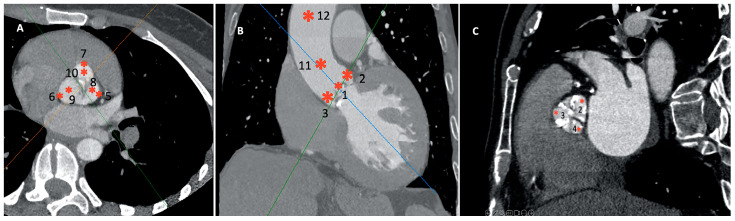
Measurement regions in the CCTA. Regions are as follows: 1: 4–5 mm above the opening of the aortic valve; 2–4: junction of the leaflets and the fibrotic anulus (left, right, and non-coronary); 5–10: mid-level of the sinus of Valsalva at the most lateral and center point of left, right, and non-coronary; 11: sinotubular junction (STJ); and 12: 4 cm from the SJT. (**A**,**C**) are oblique, (**B**) is the frontal view. From (**C**), crosshair is removed for better view. (CCTA: cardiac computed tomography angiography).

The contrast densities were measured by both an expert radiologist and an imaging cardiologist in a blinded fashion with an AW Server workstation (GE Healthcare, Boston, MA, USA) in Hounsfield units (HU) in two sets of regions:

1. Twelve distinct regions in 3D reconstruction mode: 4–5 mm above the opening of the aortic valve (OAV); at the junction of the leaflets and the fibrotic annulus (left, AL; right, AR; and non-coronary, AN); the mid-level of the sinus of Valsalva at the most lateral (Valsalva lateral left, VLL; Valsalva lateral right, VLR; and Valsalva lateral non-coronary, VLN) and at the mid-point (Valsalva center left, VCL; Valsalva center right, VCR; and Valsalva center non-coronary, VCN); and in the midline of the sinotubular junction (STJ) and 4 cm from the SJT (Figure 2). The region of interest was 3–5 mm^2^. Patients with a high grade of the beam-hardening effect due to severe calcification limiting the evaluation were excluded.
2. Right and left ventricular outflow tract (RVOT/LVOT): 2–4 mm below the pulmonary and aortic valve in the centerline of the outflow tracts, respectively.

Four pathways were defined for the evaluation of results:

1. Density differences of the perivalvular regions in both group 1 and 2, independently.
2. Density differences between the two groups for each region.
3. Possible correlation between the ECHO and the CCTA density parameters in SAS patients.
4. Possible effects of demographic data on CCTA densities.

All parts of the research complied with all the regulations of institutional ethical committee permission: DE RKEB/IKEB 6284/2022.

### Statistical Considerations

Statistical analysis was performed using SPSS version 22.0 (IBM, Armonk, NY, USA) software. Data were expressed as the mean ± SD for continuous variables. The distribution of variables was evaluated by the Kolmogorov–Smirnov test. Differences between the groups were evaluated by the Mann–Whitney test. Interobserver variability in the CCTA measurements was expressed by the intraclass correlation coefficient (ICC) and Bland–Altman plots. The correlations were determined by Spearman’s analysis. *p* values < 0.05 were considered significant. Specificity, sensitivity, receiver operating characteristic curve (ROC), area under the curve (AUC), and Youden index were calculated by MedCalc version 13.3.3.0 (MedCalc Software Ltd, Ostend, Belgium).

## 3. Results

Demographics are presented in Table 1. As was expected, the stenotic valve group patients were significantly older and the group had a higher number of males, both known characteristics of senile aortic valve stenosis. With regard to hypertension, diabetes mellitus, dyslipidemia, previous pacemaker implantation, coronary intervention or bypass surgery, and atrial fibrillation, there were no significant differences between the two groups. Ejection fraction results were fairly similar, mostly in the normal range. Higher than grade 2 aortic regurgitation was more common in the stenotic group, which was due to the non-properly closing diseased aortic valve. No or mild regurgitation was present mostly in both groups but still offered significantly higher values in group 1 (0.875 ± 1.018 vs. 0.077 ± 0.277, *p* = 0.006).

**Figure 3:**
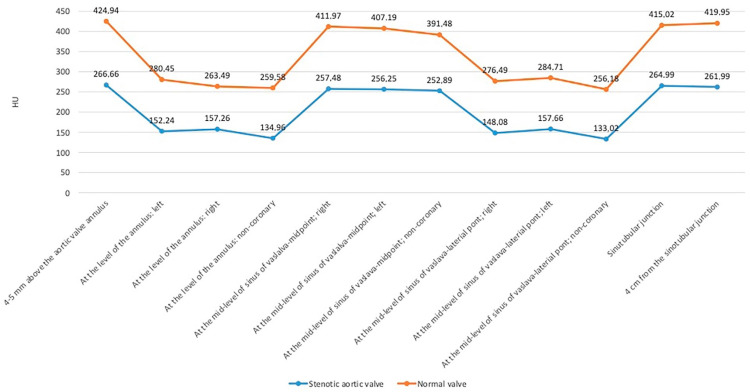
Average densities from both groups are shown in a graphical presentation. For all regions, higher densities are measured in the normal valve group.

Table 2 and Figure 3 show the contrast densities in all 12 regions for both groups. Regardless of whether the density was measured in the normal or stenotic valve, in the peri-jet regions, i.e., above the aortic valve opening, at the mid-level center of each sinus of Valsalva, at the STJ, and at 4 cm from the STJ, the HU was significantly higher than in the remote areas, i.e., at the junction of the leaflets at the level of the annulus and at every mid-level lateral point of the sinus of Valsalva. Additionally, in the normal valve group, in every region, the densities were significantly higher than in the stenotic valve group.

**Figure 4:**
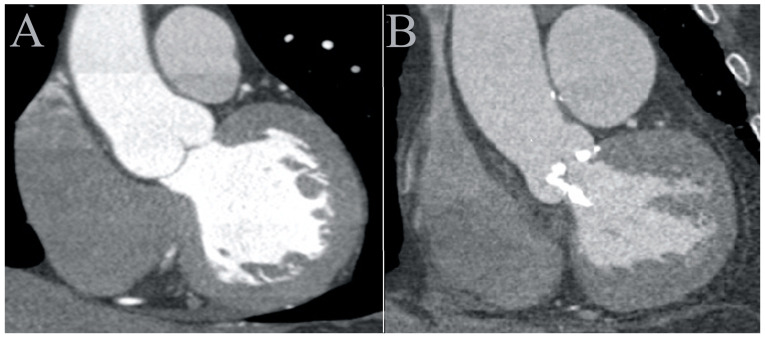
Frontal CCTA images (**A**) and (**B**) from a normal and stenotic aortic valve patient, respectively. Brighter (higher HU) contrast accumulation is evident in the normal valve patients in all regions. CCTA: cardiac computed tomography angiography; HU: Hounsfield unit.

Figure 4 presents two CCTA images in the frontal plane, with “A” and “B” representing a normal and a stenotic valve patient, respectively. Without measuring HU, it is clear that the densities in all regions are higher in the normal valve image.

Table 3 presents data investigating the consistency and agreement between the densities measured by two different experts. Interclass correlation coefficient calculation showed significant agreement between the investigators in all regions. Bland–Altman plot results proved that apart from measurements at the non-coronary annulus, mid-level of right, and non-coronary sinus of Valsalva lateral points, where the 95% limit of agreement is completely off 0, good agreement was found between the two investigators. When evaluating the bias, it is visible that apart from the non-coronary annulus and at 4 cm from the STJ, one investigator always measured higher values.

As shown in Table 4, in terms of the correlation between the ECHO and the CCTA, good correlation was found between the AVA and the densities at the AR (R = −0.366, *p* = 0.020), AL (R = −0.320, *p* = 0.044), and AN (R = −0.300, *p* = 0.060); VLR (R = −0.430, *p* = 0.006) and VLN (R = −0.300, *p* = 0.060); VMR (R = −0.430, *p* = 0.006), and VCN (R = −0.535, *p* < 0.001); and at the STJ (R = −0.399, *p* = 0.011) and the 4SJT (R = −0.442, *p* = 0.004); only the AAV, VLL, and VCL showed no correlation. In the case of the AN and the VLR, a reasonable correlation was found between all ECHO and CCTA parameters (AN vs. MG: R = 0.349, *p* = 0.027; AN vs. PG: R = 0.332, *p* = 0.037, AN vs. V_max_: R = 0.341, *p* = 0.031; AN vs. AVA: R = −0.300, *p* = 0.060; VLR vs. MG: R = 0.448, *p* = 0.004; VLR vs. PG: R = 0.355, *p* = 0.024; VLR vs. V_max_: R = 0.412, *p* = 0.008; VLR vs. AVA: R = −0.407, *p* = 0.009). LVEF and aortic regurgitation had no effect on the densities. With regard to RVOT densities, no difference was found between the two groups: 151.93 ± 58.33 vs. 144.67 ± 65.20, *p* = 0.607. In the case of LVOT, a significant difference was detected with a higher HU in the case of stenotic valves: 374.36 ± 118.99 vs. 269.48 ± 61, *p* = 0.001.

Table 5 presents the sensitivity, specificity, Youden index, and AUC for each region’s cut-off value. The highest Youden index was at the level of annulus in the right cusp and the lowest at the mid-level of the left sinus in the lateral point: 0.8167 and 0.6833, respectively. The AUC was highest at the level of annulus in the right cusp and lowest at the mid-level of the non-coronary sinus in the center point: 0.930 and 0.857, respectively. *p* values at cut-off densities in all regions proved to have a significant prognostic value.

## 4. Discussion

Reviewing the literature on aortic stenosis diagnostics and hemodynamics along with consultation with fluid dynamic specialists, the hypothesis was formed that increased blood velocity through the severely stenotic aortic valve should have an influence on the contrast densities in the perivalvular region, and that these density changes might correlate with the parameters used in ECHO to define the severity of aortic valve stenosis. Based on our findings, the densities in all perivalvular regions are always higher in normal aortic valve situations than in severely stenotic cases. Moreover, regardless of a normal or diseased valve, the densities are always higher in the central peri-jet areas than in the lateral regions. Lastly, the densities in certain regions correlated well with ECHO values used for grading aortic valve stenosis, and vice versa, the value of AVA had a significant correlation with density measurements in almost all perivalvular regions.

To understand the findings above, the literature was thoroughly investigated, and several fluid dynamic models have successfully investigated the flow and pressure changes in the stenotic aortic valve [16]. Based on these in vivo and vitro measurements, in the case of a normal aortic valve, usually no energy transformation takes place when the blood flows from the left ventricle to the ascending aorta through the aortic valve; since the velocity and the pressures do not change significantly, the pressure recovery is almost at full scale. When AVA decreases, the jet flow intensifies, and the kinetic energy of the flow increases. As the blood reaches the lateral parts of the sinus of Valsalva and the arc of the ascending aorta, the flow decelerates, and partial pressure (static) energy recovery takes place. To define the role of the stenotic valve in the total pressure drop, the valve resistance index (IVR) can be calculated based on the ratio of the pressure loss due to the stenotic valve over the total pressure loss. Traeger et al. suggested that an AVA of 0.9 cm^2^ can result in an IVR of 0.9; in other words, 90% of the pressure drop is due to the stenotic valve [17]. Hoeijmakers et al. created a workflow using computational fluid dynamics (CFD) to calculate the valve resistance index, which quantifies the contribution of the stenotic aortic valve to the transvalvular pressure drop, offering help when the geometric and dynamic ECHO measurements are conflicting [18].

Taking into consideration the above-mentioned hemodynamic observations, it can be understood that in the peri-jet area with every systole, the flow increases, and high-density regions quickly form, whereas in the remote/lateral perivalvular regions, where the flow decelerates, a slower increase in density will take place. The difference between normal and stenotic valve densities having higher values earlier might be due to a faster buildup of contrast material when the flow is not obliterated by the stenotic aortic valve. It is important to mention that since the injections of the contrast material are for only 10–15 s, the density values change from moment to moment. Therefore, we can anticipate that in in vitro models, after constant injection of contrast material, all these differences would diminish over time, and a steady-state situation would evolve.

Additionally, the question may arise as to whether the different injection parameters caused these differences in the above-mentioned comparison between the two groups. Lell et al. compared the enhancement from the pulmonary trunk to the branches of the aorta with different injection protocols, using the variables of speed, the amount of contrast media (using iodine delivery rate, IDR), and the kV (energy of photons used in the CCTA). Based on their results, there were no significant enhancement differences in the pulmonary trunk. In the ascending aorta, the protocol using the highest IDR with the lowest kV (70) resulted in a significantly higher HU, with an average difference of approximately 120–130 HU [19]. In our investigation, we used the same kV of 100, far from the K-edge of iodine, with different IDRs (0.75 g/s vs. 1.5 g/s in the stenotic vs. normal valves, respectively), resulting in approximately 130 and 150 HU differences between the stenotic and normal valves (higher baseline HU for the latter), with a greater difference in the peri-jet areas. From this, we assume that even using the same injection protocol (same kV and IDR) for both groups, these differences would still exist.

Additionally, it is important to mention that in the RVOT region, there was no density difference between the two groups, probably because dilution had already started, making the HU close to the normal blood value. Other explanations may include that pulmonary circulation dispenses the pressure buildup and contrast material accumulation due to the flow limiting severe aortic stenosis. In the case of the LVOT, there was a significantly higher contrast density in the stenotic valve group, which explains the lower peri-aortic values in this group.

When evaluating the correlations between the ECHO and the CCTA measurements, interestingly, only at the mid-level lateral of the right sinus of Valsalva did we find a significant correlation between the density and all four parameters measured by the ECHO. The explanation behind that is the fact that the greater curve of the ascending aorta is on the right, and the jet heads towards the right wall of the aorta, the circulation zone in this region being more stable than on the other side of the jet [18].

In the peri-jet regions of SJT and 4 cm above the latter regions, the density values were still high, being moderately far from the highest velocity of the blood at the opening of the aortic valve. Since the speed of the blood is at least 4 m/s, in these regions, the speed probably does not decrease significantly; thus, the energy transformation in the peri-jet regions does not evolve to a magnitude that would change the contrast enhancement compared to the highest velocity region above the valve opening.

Interestingly, from the ECHO parameters, only AVA had a significant correlation with almost all the densities. One of the possible explanations for this is the structural difference in the population with degenerated aortic valves. The density and the distribution of calcium deposits inside the leaflets change from patient to patient. This fact results in variations between the orientation of the turbulent flow coming from the ventricle, producing inconsistent ECHO values, mainly in the case of the V_max_ and the maximal gradient, while the AVA is a calculated parameter generated from the LVOTVI and AOV VTI with less patient-to-patient variation. Reviewing the literature, we did not find any exact papers discussing a standard calcification pattern of leaflets of the aortic valve. In our patient population, all patients had a tricuspid aortic valve with all three cusps moving. Evaluating the valves with CCTA for calcification, we could only conclude that all cusps were involved in the calcification process to different levels but were not able to quantify the differences. Calcium scoring would be a possible way to gain this information.

With regards to the efficacy of this new diagnostic method, based on the sensitivity and specificity values at the best cut-off densities, the Youden index was always above 0.6833, which offers good coupling of sensitivity and specificity in all regions. Having these values in hand, the AUC was also high in all regions. For the last few years, the importance of aortic valve calcification correlation with echocardiography, or even for prognostic evaluation, has been gaining strength rapidly. Numerous smaller and larger investigations found very good performance compared to echocardiography as the gold standard, even in different patient subsets, mostly focusing on concordant severe aortic stenosis patients. Table 6 presents studies with the highest sample number and similar patient materials and investigation methods. When compared to Table 5 containing our findings, the results are similar and comparable, suggesting the new method’s possible feasibility in the evaluation of aortic valve stenosis [20,21,22,23,24].

With all the information discussed above, a question might arise regarding the everyday use, the density differences, and the correlations found. As mentioned above, in one-third of the cases, the diagnosis of severe aortic stenosis is unclear, or the values measured with ECHO are conflicting, i.e., low-flow and low-gradient situations. In these borderline cases, CCTA parameters, with excellent reproducibility, can help differentiate between severe and non-severe stenosis, especially when cardiac MRI is not available to clarify the issue [25,26]. Also, using the density values correlating with one or all ECHO parameters, the grade of aortic valve stenosis can be evaluated when CCTA is performed for reasons other than for pre-TAVI investigation. Even with the findings above, it should be mentioned that ECHO is still the most inexpensive, fastest, most easily accessible, very sensitive, and most specific method for the diagnosis and follow-up of aortic stenosis.

When interpreting all the different statistical measurements of the regions, we see slight differences for several in favor. For the region 4–5mm above the valve opening and all values for the correlation, ICC, AUC, sensitivity, specificity, and AUC, better than average results were observed. The reason for this might be that the blood column of the jet does not experience changes due to geometrical stability (i.e., flow separation), thus providing the highest similarity in all patients. Based on the above, using the contrast density values from this region for valve stenosis grading might be the most optimal from all densities.

### Limitations

First, the low sample number was one of the major limitations of the investigations. External validation would also strengthen the usefulness of the new method. Recruiting more patients with a 20% RR interval reconstruction was limited since ensuring ALARA (an as low as reasonably achievable radiation dose without a significant loss of image quality) principles means prospective ECG triggering should be used. New and improved CCTA techniques can overcome the above issue with low effective radiation with systolic and diastolic acquisition. Second, normal and stenosis aortic values were obtained and compared with different contrast administration protocols. Even though the literature shows that different protocols might not change the enhancement, to be objective, the same kV and IDR should be used when an investigation is performed. Third, due to the cubital vein not always being available, other distal venous access points from the lower arm (or even from the other arm) with smaller lumen lines need to be used, which might have an effect on densities due to the longer route to the heart, along with increased distribution volume; these must be further investigated with the expertise of fluid dynamic specialists. Fourth, special cases of borderline aortic stenosis, i.e., low-flow, low-gradient situations, and their effect on densities were not investigated in the study. Fifth, radiation is always a concern with CCTA, especially when a head-to-head comparison is made with a diagnostic method not using ionizing radiation (ECHO). Recent dose-reduction techniques can bring the effective radiation down to 1–3 mSv, comparable to the yearly background radiation from sea level to 1000 m, decreasing the risk of medical-radiation-induced cancer. Sixth, our protocol did not include an acquisition phase dedicated to Ca-score measurements of the aortic valve. In the future, measuring both Agatston score and the densities, or even combing the power of both, might give even better predictive value to the evaluation of the aortic valve stenosis significance by CCTA.

## 5. Conclusions

Perivalvular contrast densities using CCTA decrease as the severity of aortic stenosis increases, and numerous density values are in good correlation with the gold standard values of ECHO. CCTA could serve as an auxiliary imaging method when the severity of aortic valve stenosis is unclear. Also, patients with a CCTA for an indication other than pre-TAVR investigation who show aortic valve calcification, or a bicuspid aortic valve, could have the grade of aortic stenosis evaluated based on perivalvular densities. Along with the above-mentioned results, it must be emphasized that data from images should always be interpreted with the patient’s symptoms. ESC/EACTS guidelines on aortic stenosis highlight the importance of correlating symptoms even in questionable/borderline-significance aortic valve stenosis [27].

## Figures and Tables

**Table 1:** Baseline clinical characteristics of both groups.

	Group 1	Group 2	*p*
Age (years)	79.33 ± 6.32	57.00 ± 10.47	<0.001
Gender (male/female)	24/16 (60/40%)	3/12 (20/80%)	0.014
Hypertension	34 (85%)	9 (60%)	0.068
Diabetes mellitus	14 (35%)	3 (20%)	0.344
Dyslipidemia	12 (30%)	5 (33%)	1.000
Pacemaker implantation	6 (15%)	1	0.660
Coronary intervention or bypass operation	14 (35%)	2	0.184
Atrial fibrillation	12 (30%)	1 (6%)	0.086
Ejection fraction (%)	50.25 ± 8.90	54.53 ± 7.05	0.093
Aortic regurgitation	0.875 ± 1.018	0.077 ± 0.277	0.006
Grade 0	20	14	
Grade 1	8	1	
Grade 2	9	0	
Grade 3	3	0	

In the stenotic valve group, patients were significantly older, and the number of male patients was higher. Grade 2 or above regurgitation was more common in the diseased group, resulting in a significantly higher grade of valve insufficiency. Values are means ± SD or percentages of subjects; *p* for significance.

**Table 2:** Density values in Hounsfield units in all 12 areas for both groups.

	Group 1	Group 2	*p*
4–5 mm above the valve opening	266 ± 58	424 ± 91	<0.001
At the level of the annulus right	152 ± 48	280 ± 65	<0.001
left	157 ± 50	263 ± 72	<0.001
non-coronary	134 ± 49	259 ± 63	<0.001
At the mid-level of the sinus of Valsalva—midpoint right	257 ± 60	411 ± 88	<0.001
left	256 ± 60	407 ± 94	<0.001
non-coronary	252 ± 62	391 ± 89	<0.001
At the mid-level of the sinus of Valsalva—lateral point right	148 ± 47	276 ± 78	<0.001
left	157 ± 52	284 ± 83	<0.001
non-coronary	133 ± 42	256 ± 87	<0.001
Sinotubular junction	264 ± 54	415 ± 87	<0.001
4 cm from the sinotubular junction	261 ± 55	419 ± 94	<0.001

In all regions, significant density differences were found between the groups, with higher values in the normal valve condition. Values are means ± SD of subjects; *p* for significance.

**Table 3:** Interobserver variability between the densities measured by two different experts with consistency of the data.

	ICC	*p*	Bias	SD of Bias	95% Limits of Agreement	
					from	to
4–5 mm above the valve opening	0.878	<0.001	5.38	42.19	−77.31	88.07
At the level of the annulus right	0.455	<0.001	94.03	56.89	−17.46	205.50
left	0.623	<0.001	75.31	42.05	−7.11	157.70
non-coronary	0.411	<0.001	108.40	47.48	15.31	201.40
At the mid-level of the sinus of Valsalva—midpoint right	0.930	<0.001	−0.60	30.63	−60.62	59.43
left	0.947	<0.001	6.12	25.67	−44.20	56.45
non-coronary	0.874	<0.001	15.09	42.14	−67.50	97.68
At the mid-level of the sinus of Valsalva—lateral point right	0.421	<0.001	96.96	43.78	11.16	182.80
left	0.382	<0.001	89.46	46.62	−2.308	181.2
non-coronary	0.371	<0.001	102.6	44.89	14.61	190.60
Sinotubular junction	0.978	<0.001	1.95	17.22	−31.80	35.70
4 cm from the sinotubular junction	0.981	<0.001	−6.40	13.69	−33.24	20.43

Left two columns present ICC and *p* values; right four columns provide Bland–Altman plot results. At least acceptable and, in certain cases, good interobserver reliability was found in almost all regions. ICC: interclass correlation coefficient; *p* for significance; SD: standard deviation.

**Table 4:** Correlation between the contrast densities and the ECHO values.

	MG (mmHg)	PG (mmHg)	V_max_ (m/s)	AVA (cm^2^)	LVEF (%)	Aortic Regurgitation
4–5 mm above the valve opening	R = 0.073	R = 0.034	R = 0.035	R = −0.302	R = 0.136	R = 0.167
	*p* = 0.652	*p* = 0.837	*p* = 0.829	*p* = 0.058	*p* = 0.404	*p* = 0.303
At the level of the annulus	R = 0.164	R = 0.149	R = 0.239	R = −0.366	R = −0.168	R = 0.251
right	*p* = 0.311	*p* = 0.359	*p* = 0.137	*p* = 0.020	*p* = 0.301	*p* = 0.118
left	R = 0.050	R = −0.015	R = −0.048	R = −0.320	R = −0.119	R = 0.372
	*p* = 0.760	*p* = 0.927	*p* = 0.768	*p* = 0.044	*p* = 0.463	*p* = 0.018
non-coronary	R = 0.349	R = 0.332	R = 0.341	R = −0.300	R = 0.023	R = 0.263
	*p* = 0.027	*p* = 0.037	*p* = 0.031	*p* = 0.060	*p* = 0.890	*p* = 0.101
At the mid-level of the sinus of Valsalva—center	R = 0.127	R = 0.123	R = 0.197	R = −0.430	R = 0.207	R = 0.093
right	*p* = 0.434	*p* = 0.449	*p* = 0.227	*p* = 0.006	*p* = 0.201	*p* = 0.567
left	R = 0.058	R = 0.057	R = 0.075	R = −0.216	R = 0.192	R = 0.190
	*p* = 0.724	*p* = 0.728	*p* = 0.647	*p* = 0.180	*p* = 0.236	*p* = 0.240
Non-coronary	R = 0.240	R = 0.210	R = 0.247	R = −0.523	R = 0.255	R = −0.013
	*p* = 0.135	*p* = 0.193	*p* = 0.124	*p* = 0.001	*p* = 0.113	*p* = 0.937
At the mid-level of the sinus of Valsalva—lateral point	R = 0.448	R = 0.355	R = 0.412	R = −0.407	R = −0.076	R = 0.273
right	*p* = 0.004	*p* = 0.024	*p* = 0.008	*p* = 0.009	*p* = 0.642	*p* = 0.088
left	R = 0.072	R = 0.053	R = −0.026	R = −0.224	R = 0.131	R = 0.220
	*p* = 0.661	*p* = 0.744	*p* = 0.873	*p* = 0.165	*p* = 0.421	*p* = 0.173
Non-coronary	R = 0.269	R = 0.253	R = 0.243	R = −0.535	R = −0.030	R = 0.136
	*p* = 0.093	*p* = 0.116	*p* = 0.131	*p* < 0.001	*p* = 0.854	*p* = 0.404
Sinotubular junction	R = 0.072	R = 0.083	R = 0.130	R = −0.399	R = 0.219	R = 0.092
	*p* = 0.657	*p* = 0.611	*p* = 0.425	*p* = 0.011	*p* = 0.176	*p* = 0.574
4 cm from the sinotubular junction	R = 0.166	R = 0.139	R = 0.181	R = −0.442	R = 0.324	R = 0.165
	*p* = 0.305	*p* = 0.393	*p* = 0.265	*p* = 0.004	*p* = 0.042	*p* = 0.307

In almost all regions, a significant correlation was found between the densities and the aortic valve opening area. The densities at the level of the non-coronary annulus and at the mid-level lateral point of the right sinus of Valsalva showed good correlation with all ECHO parameters used for the evaluation of the aortic valve stenosis. Left ventricular ejection fraction and aortic regurgitation had no effect on densities. MG: mean gradient; PG: peak gradient; Vmax: maximum velocity; AVA: aortic valve opening area; LVEF: left ventricular ejection fraction; *p* for significance; SD: standard deviation; ECHO: echocardiography; *p* for significance.

**Table 5:** Performance measurements for each region.

	AUC	*p*	Sensitivity (%)	Specificity (%)	Youden Index	Cut-Off (HU)
4–5 mm above the valve opening	0.897	<0.001	97.5	80	0.7750	377
At the level of the annulus right	0.930	<0.001	95.0	86.67	0.8167	236.3
left	0.877	<0.001	92.5	80.0	0.7250	221.9
non-coronary	0.915	<0.001	90.0	86.67	0.7667	206.8
At the mid-level of the sinus of Valsalva- center point right	0.892	<0.001	95.0	80.0	0.7500	357.5
left	0.884	<0.001	85.0	86.67	0.7167	319.4
non-coronary	0.857	<0.001	90.0	80.0	0.7000	339.1
At the mid-level of the sinus of Valsalva- lateral point right	0.887	<0.001	92.5	80.0	0.7250	211.6
left	0.895	<0.001	95.0	73.33	0.6833	231.8
non-coronary	0.902	<0.001	90.0	80.0	0.7000	180.2
Sinotubular junction	0.887	<0.001	92.5	80.0	0.7250	346.3
4cm from the sinotubular junction	0.920	<0.001	92.5	80.0	0.7250	334.3

In all regions, high sensitivity, specificity, and AUC values were measured at the cut-off values. For all cut-off densities, the level of prediction was significant. AUC: area under the curve; *p* for significance; HU—: Hounsfield unit.

**Table 6:** Performance measurements in investigations for aortic valve calcification.

	AUC		Sensitivity (%)		Specificity (%)	
	Women	Men	Women	Men	Women	Men
Katagiri et al.	0.93	0.88	91.50	84.80	89.30	83.30
Pawade et al.	0.92	0.89	87	80	84	82
Messika-Zeituon et al.	0.89		93		83	
Clavel et al.	0.91	0.90	86	89	89	80
Ouchi et al.	0.957	0.955	87.10	84.60	93.20	97.10

For each investigation, aortic valve calcification compered to gold standard echocardiography in concordant severe aortic valve stenosis proved good performance. AUC: area under the curve.

## Data Availability

All data are available at request from the corresponding author after permission is sought from the local institutional ethical authority.

## Abbreviations

4STJ	4 cm from the sinotubular junction at the midline
AAV	4–5 mm above the aortic valve
AL	junction of the left leaflet and the fibrotic annulus
AN	junction of the non-coronary leaflet and the fibrotic annulus
AR	junction of the right leaflet and the fibrotic annulus
AoVVTI	aortic valve velocity time integral
AVA	aortic valve area
CCTA	cardiac computed tomography angiography
ECHO	echocardiography
LVEF	left ventricular ejection fraction
LVOT	left ventricular outflow tract
MG	mean transvalvular gradient
LVOT VTI	left ventricular outflow tract velocity time integral
LVOTd	left ventricular outflow tract diameter
OAV	opening of the aortic valve
PG	peak transvalvular gradient
RVOT	right ventricular outflow tract
SAS	severe aortic stenosis
SAVR	surgical aortic valve replacement
STJ	midline of the sinotubular junction
TAVR	transcatheter aortic valve replacement
VLL	mid-level of the left sinus of Valsalva at the most lateral point
VLN	mid-level of the non-coronary sinus of Valsalva at the most lateral point
VLR	mid-level of the right sinus of Valsalva at the most lateral point
V_max_	maximum velocity above the opening of the aortic valve
VCL	mid-level of the left sinus of Valsalva in the center
VCN	mid-level of the noncoronary sinus of Valsalva in the center
VCR	mid-level of the right sinus of Valsalva in the center
